# The Role of Vitamin E in Slowing Down Mild Cognitive Impairment: A Narrative Review

**DOI:** 10.3390/healthcare9111573

**Published:** 2021-11-18

**Authors:** Ram Lakhan, Manoj Sharma, Kavita Batra, Frazier B. Beatty

**Affiliations:** 1 Department of Health and Human Performance, Berea College, Berea, KY 40404, USA; Lakhanr@berea.edu; 2 Department of Social & Behavioral Health, School of Public Health, University of Nevada, Las Vegas, NV 89119, USA; manoj.sharma@unlv.edu; 3 Office of Research, Kirk Kerkorian School of Medicine, University of Nevada, Las Vegas, NV 89102, USA; 4 Graduate Department of Public Health, School of Health Sciences, Regis College, Weston, MA 02493, USA; frazier.beatty@regiscollege.edu

**Keywords:** dementia, mild cognitive impairment, vitamin E, amnesia

## Abstract

With the aging population, dementia emerges as a public health concern. In 2012, the Health and Retirement Study found that 8.8% of adults over 65 years suffered from dementia. The etiopathogenesis and treatment of dementia are not well understood. Antioxidant properties of Vitamin E and its major elements tocopherols and tocotrienols have been reported to be effective in slowing down the progression of dementia from its initial stage of Mild cognitive impairment (MCI). Therefore, the current review aims to explore the role of vitamin E on MCI. A literature search using the key words “Vitamin E, tocopherols, tocotrienols, and mild cognitive impairment” was conducted in MEDLINE (PubMed), CINAHL, and Google Scholar. The inclusion criteria were: (1) articles published in the past ten years; (2) published in English language; (3) published in peer-reviewed journals; and (4) descriptive and epidemiological or evaluation studies. Articles published prior to 2010, focused on other forms of dementia than MCI, grey literature and non-peer-reviewed articles were excluded. A total of 22 studies were included in the narrative synthesis. The results were equivocal. Eleven studies showed some level of the neuroprotective effect of Vitamin E, tocopherols and tocotrienols on the progression of MCI. The mixed results of this review suggest further exploration of the possible protective effects of Vitamin E on the development of dementia. Future studies can be conducted to decipher antioxidant properties of vitamin E and its association with slowing down the cognitive decline.

## 1. Introduction

Dementia is a serious public health concern with nearly 50 million people having some form of dementia globally [1]. Reportedly, about 60% of the dementia population live in low and middle-income countries (LMICs) [1]. Estimates suggest that about 10 million people get dementia every year and about 15–20% of elderly population reported having mild cognitive impairment (MCI) as the early stage of dementia [1,2,3]. MCI causes a slight but observable and measurable decline in the memory and thinking skills of an individual. In some individuals, MCI can be reversible (if physiological in origin), however, the likelihood of the reversal to the normal cognitive capability is less for majority of people if it is pathological [2,3]. With due course, MCI advances to the next stage with nearly 65% of people developing more severe forms [2,3].

According to the previous meta-analytical evidence presented by the American Academy of Neurology, the prevalence of MCI was nearly 7% among people with age 60–64 years, which increases with advancing age [3,4]. In the United States (U.S.), the highest prevalence was reported among elderly above 75 years of age [4]. These trends are not only limited to developed countries, low- and middle-income countries indicate similar patterns [5]. However, due to lack of population-based studies, the true estimates are unavailable. According to the previous reports, the prevalence of MCI ranged from 4.5% to 15.4% among South Asian countries [6,7,8,9]. Differences in MCI by demographic characteristics (e.g., gender, race/ethnicity, education) were also noted with women being at greater lifetime risk for dementia compared to their male counterparts [10,11,12]. The occurrence of MCI is found delayed and lenient towards the end of life among whites and highly educated people while the onset of MCI is observed at younger age among blacks and those with lower education attainment [10,11,12]. The lifetime risk of dementia is 21% among men with an associate degree while it is 35% for those who have less than high school education [10,11,12]. White women have a shorter cognitively impaired life compared to black women (6 years vs. 12 to 13 years) [10,11,12]. The burden of dementia further translates into higher cost associated with its management [2]. According to the Alzheimer association, through identification of early stages of Alzheimer disease (AD) i.e., MCI, nearly $7 to $7.9 trillion in health and long-term care can be saved [2].

MCI has severe implications for the patients and their family members and challenges are multifactorial in origin [13]. Often time patients and their family members are unable to identify cognitive decline at earlier stages, particularly in older population groups, in whom cognitive decline is a normal physiological phenomenon. Moreover, cooccurrence of other age-associated diseases are likely to occur in this group with a limited ability to make a differential diagnosis [14,15]. Cognitive insufficiency impacts the quality of life, individual’s functioning, their relationship with the family members, and their self-esteem [14,15]. Caregivers experience high level of caregiving burden for the larger population of MCI [16]. Given the unavailability of medication to treat, prevent, or slow the progression of MCI to dementia, preventive strategies take precedence for at-risk population groups to prevent progressive deficits [17]. Prevention of somatic diseases, promotion of physical and mental exercise, cognitive training, avoidance of toxins, reduction in stress, stopping smoking, and use of dietary compounds such as antioxidants and supplements are some of the suggested to address MCI [17,18]. Among antioxidants, vitamins play a critical role in reducing or delaying to the process of cognitive decline in people with MCI. Among all vitamins, vitamin E was found to be effective in reducing MCI [17,18,19,20,21,22,23]. Vitamin E is a fat-soluble vitamin and found in variety of foods [19]. Its usable form (i.e., alpha tocopherol) is considered a scavenger of free radicals in the body [19], which controls brain prostaglandin synthesis and regulates nucleic acid synthesis. While some studies have documented association of vitamin E intake in slowing down the progression of MCI, collective evidence to investigate its significance is still lacking [17,18,19,20,21,22,23]. Therefore, the purpose of this study was to review existing literature to decipher role of vitamin E in slowing down MCI progression.

## 2. Methods

### 2.1. Search Strategy

Bibliographical databases, including Medline (PubMed), CINAHL, and Google Scholar were quickly searched in January/February 2020. Pharmacological synonyms of vitamin E were used as related terms to locate potential evidence to be included in this review. Articles related to cognitive impairment were also sought using the Boolean operator “AND” to narrow down search results to include articles containing the specified terms. A detailed list of key words is shown in Table 1.

### 2.2. Inclusion Criteria and Data Abstraction

The inclusion criteria of this review were the following; (1) observational, randomized controlled trials, clinical and laboratory studies published over past ten years; (2) studies published in English language; (3) published in peer-reviewed journals; and (4) descriptive and epidemiological or evaluation-based studies. The exploration of preventive relationship of vitamin E with mild cognitive impairment is relatively new. Therefore, we selected to assign a broad range of criteria including human and animals-based studies published in the 10 years of timeframe. Articles published before 2010, focused on other forms of dementia than MCI, grey literature, abstracts—only studies, and non-peer-reviewed articles were excluded. We also conducted a post-hoc search in July 2021 to update our literature matrix used for this review. Details about the search results were saved in the spreadsheet by the lead author. Titles and abstracts were screened for relevancy and eligibility. If found relevant, full-texts were read thoroughly and data were extracted in a standardized data collection form. Variables such as year of publication, study type, outcomes, key findings, neuroprotective role of vitamin E, and conclusions were tabulated in the data collection form.

## 3. Results

**Figure 1:**
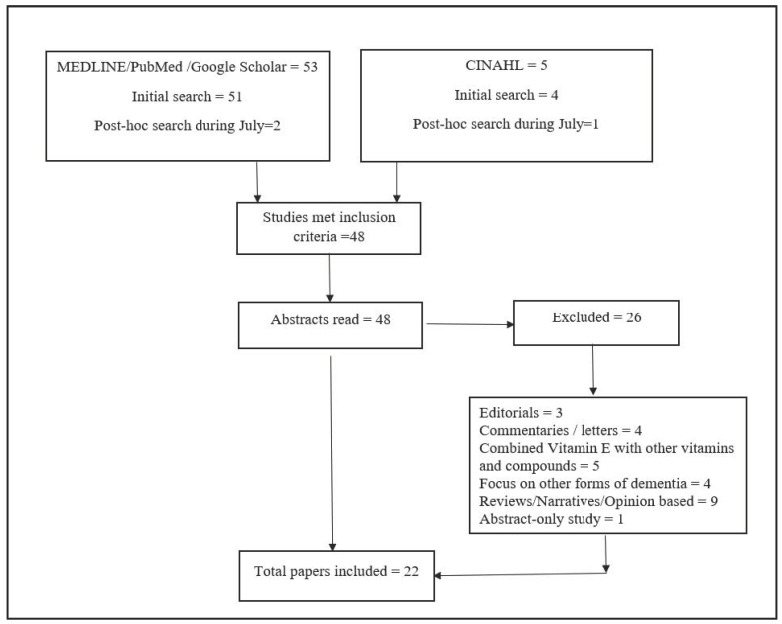
Flow diagram of the literature search and selection process.

Following keyword search, 53 articles in Medline/PubMed/Google Scholar and 5 in CINAHL database were found. Out of those, 48 studies met the inclusion criteria. Abstracts of all 48 studies were screened. Twenty-six studies were excluded for variety of reasons of being editorials, commentaries/letters, abstract-only study, combined vitamin E with other vitamins, focused on other form of dementia rather than MCI, reviews, narratives and opinion-based papers. Finally, 22 papers [19,20,21,22,23,24,25,26,27,28,29,30,31,32,33,34,35,36,37,38,39,40,41] were included in this review for the data summarization (Figure 1). Characteristics of finally included studies are provided in Table A1 in Appendix A.

Of 22 finally included studies, seven were conducted with animals. Out of seven, six studies conducted on rats, mice, and other animals exhibited some level of the neuroprotective effect of vitamin E by lowering the rate or delaying cognitive impairment progression. Similarly, out of 15 studies conducted on humans, eight studies reported Vitamin E’s role in lowering the risk or delaying cognitive impairment. Two studies, one cohort and a double-blind randomized placebo-controlled study, demonstrated Vitamin E’s effect in improving learning and memory functions. Two studies of each experimental and double-blind randomized placebo-controlled did not find any effect of Vitamin E on cognitive impairment while a clinical study14 suggested a potentially favorable effect (Table 2 and Table A1).

## 4. Discussion

The purpose of this review was to examine the effect of Vitamin E in slowing down cognitive decline in MCI. Some evidence from our literature synthesis points to the putative role of Vitamin E in slowing down MCI progression to dementia. Overall, the review collectively demonstrated through analysis of various experimental studies in rats, mice, and animals and cross-sectional, case-control, prospective cohort, experimental, clinical, and double-blind randomized in human that Vitamin E has some neuroprotective effect in slowing down progression to dementia. One clinical study carried out to lower the effect of cisplatin chemotherapy neurotoxicity, the supplementation with vitamin E (alpha tocopherol) has shown lower level of neurotoxicity, which indicates that vitamin E plays a neuroprotective role [42] even when the cause of neurotoxicity could be other than mild cognitive impairment. The findings of Gugliandolo et al. (2017) have reported similar physiological responses of Vitamin E on MCI [43,44,45]. Another study by Kaneai et al., indicated that vitamin E offers some neuroprotective benefits by improving neurotransmission [45]. Presently, no medication can treat, prevent, or slow the progression of MCI to dementia. However, it is important to explore the role of vitamins and antioxidants in reducing or delaying to the process of cognitive decline in people with MCI [17]. Studies show that high plasma Vitamin E levels have been associated with better cognitive performance in both ageing populations, dementia, and AD patients [42,43].

Consistent to previous studies it is understood that Vitamin E might have some therapeutic role when it comes to MCI and its progression to dementia. Since several studies in human as well as in rats and mice were MCI associated with lower level of tocopherol. Thus, it may be considered a good practice to maintain Vitamin E level through dietary sources. Vitamin E can also lead to toxicity that can be fatal in some cases which warrants careful monitoring of its levels in the aging population. The guidelines on the safe dose of vitamin E varies from 800–2000 IU/day as reported by previous studies [46,47]. Therefore, these findings should be considered rather carefully to prevent any toxic effects of vitamin E. In the meanwhile, more randomized controlled trials to further elucidate the role of Vitamin E on MCI must be conducted with larger sample sizes. Even though there are mixed results they favor a potential neuroprotective effect of Vitamin E in MCI. It would be recommended for clinical practice that the Vitamin E levels be checked annually in the elderly, and they should be provided Vitamin E supplementation in maintaining its adequate level.

### Limitations

This review has certain limitations including that research articles focused on dementia, which have explored relationship of vitamin E on mild cognitive impairment but not necessarily mentioned MCI in the title or abstract, those articles might have left from inclusion. Authors utilized phrase searching, however, due to the lack of truncation rules for some terms of dementia, some articles might have been missed. Therefore, future studies using a systematic literature review with a well-defined and peer-reviewed search strategy can be conducted. Six animal studies are included in this review and comparison were made between findings in human and animals. Comparison of effect of an element between human and animal specially on cognitive function and process is extremely complex. In simpler terms, behavioral studies to investigate cognitive decline among humans offer higher inferential benefits than those being conducted among animals due to differences in their baseline intelligence levels and capabilities. Next, studies included in this review were heterogenous in terms of type of measures used to detect the cognitive decline and these measures had varied threshold criteria. These restricted our ability to generate pooled estimates, which otherwise might have helped us to quantify the association between vitamin E and neuroprotection in the form of appropriate effect sizes (such as odds ratios). In addition, understanding trajectories of change in cognitive function will be difficult with such a heterogeneity. On the other hand, the observation of physiological process relatively easier in animal population compared to human due to ethical consideration. The findings of animal studies showing favorable outcome of vitamin E on cognition process may not be rewarding when compared to human. The database such as LILACS were not explored. Few studies included in this review has a very small sample size or their results were mixed up with some other compound which might have impacted on the conclusion of this review.

Despite the limitations, the study has implications for practice. Vitamin E supplementation should be monitored closely when in combination with Vitamin E nutrients from food sources. Other than alpha tocopherol, no other component of vitamin was found helpful in slowing the process of cognitive decline. It is not known if the other form of vitamin E or its component has negative effect on cognitive process. Therefore, caution needs to be practiced in optimizing level of vitamin E that way the other components of vitamin E do not affect the properties of alpha tocopherol and its impact on MCI. In addition, this review indicates favorable physiological and neurochemical benefits of vitamin E in protecting or delaying cognitive decline process, however, effects may be different among animals and human-beings. These differences may be due to the varied vitamin E requirements and physiological mechanisms among these groups. Therefore, further research to understand associations between vitamin E requirements, consumption, and effects at organismal levels would be critical to unfold interactions of vitamin E with body mechanisms.

## 5. Conclusions

In conclusion, epidemiological, clinical, nor other studies have provided the conclusive answer to whether or not Vitamin E slows the progression of MCI. Further research with human subjects is needed to understand the safety and efficacy of Vitamin E as a nutritional supplement to promote health ageing. Future research to understand the physiological process of alpha tocopherol on cognitive process in human beings can be conducted.

## Figures and Tables

**Table 1:** List of keywords used for literature search.

Main Term	Related Terms Used
Vitamin E	Tocopherol * OR D1 alpha tocopherol OR Preventive therapy OR tocotrienols OR Aquasol E OR Antioxidant
AND	
Mild cognitive impairment	Dementia OR Alzheimer’s disease OR Cognitive decline OR Amentia OR Mental disorder OR Paranoid Dementia OR Senile Paranoid

**Table 2:** Summary results of all studies in the review (*n* = 22).

Categories		Delay or a Lower Rate of Cognitive Decline or Neuroprotective Effect	Improved Learning and Memory Functions	May Be Effective	Suggest Further Exploration	No Effect
Animals	Rats (*n* = 3)	3				
	Mice (*n* = 2)	1			1	
	Other animals (*n* = 2)	2				
Human	Cross-sectional (*n* = 1)	1				
	Case-control (*n* = 1)	1				
	Cohort (prospective) (*n* = 3)	2	1			
	Experimental (*n* = 4)	2				2
	Clinical (*n* = 2)	2		1		
	Double-blind, randomized, placebo-controlled (*n* = 4)	1	1			2
Total (*n* = 22)		15	2	1	1	4

## Appendix A

**Table A1:** Reviewed studies and their salient findings (*n* = 22).

Authors	Year	Study Type	Sample Characteristics	Findings	Intrument Used to Screen Dementia	Conclusions
Wu et al.	2010	Experimental study in rats	Rats were fed 500 IU/Kg Vitamin E with their regular diets for four weeks before performing mild fluid percussion injury (FPI). The Vitamin E counter reacted against the effects of fluid percussion injury.	Vitamin E supplementation diet counteracts the molecular substrates underlying synaptic plasticity and cognitive function in the hippocampus.	Not Applicable	Vitamin E dietary supplementation can protect the brain against the effects of mild TBI on synaptic plasticity and cognition. Declines rate of cognitive impairment.
Huang et al.	2010	Experimental study in mice	The relationship between Vitamin E was observed with protein oxidation in mice.	Protein oxidation and nitration increased in MCI.	Not Applicable	The study suggested that the therapeutic role of vitamin E should be explored in MCI.
Alzoubi et al.	2013	Experimental, Animal study	The effect of Vitamin E against a high-fat high carbohydrate diet (HFCD) was observed. It is known that HFCD accelerates learning and cognitive impairment. In this study, the HFCD or Vitamin E was administered to animals for 6 weeks. Behavioral activities were conducted to test spatial learning and memory.	Vitamin E prevented memory impairment induced by HFCD and normalized the effect of HFCD on oxidative stress.	Not Applicable	Probably Vitamin E reduces the risk of MCI by reducing probably through normalizing antioxidant mechanisms in the hippocampus.
Giraldo et al.	2014	In vivo, mice study	The effect of Vitamin E was observed on the inhibition of p38 which prevents Aβ-induced tau phosphorylation that leads to cognitive impairment.	Vitamin E inhibited tau phosphorylation and reduced cognitive impairment.	Not Applicable	Vitamin E has a therapeutic role in protecting the decline of memory impairment.
McDougall et al.	2017	Animal experimental study	The study examined learning and memory impairment in zebrafish with vitamin E deficient and sufficient.Zebrafish fed with vitamin E for 45 days acquired sufficient vitamin E levels.	Learning ability was observed in association with vitamin E level by excluding the effect of avoidance conditioning and non-associative learning. Zebrafish with low vitamin E were found learning impaired.	Not Applicable	Study proves that vitamin E plays important role in protecting cognitive delay.
Nesari et al.	2019	Experimental study on rats	The effect of Alpha-tocopherol was evaluated in view of observing its protective effect on long-term memory impairment.	The Alpha-tocopherol reduced the passive avoidance memory performance, increased the level of malondialdehyde (MDE) and reactive oxygen specifies.	Not Applicable	Alpha-tocopherol was found to have a neuroprotective effect on memory impairment.
Mehrabadi & Sadr	2020	Experimental study on rats	The effect of vitamins D_3_ and E, in a combination of both, was observed on learning and memory. 60 rats received different doses of vitamins.	Memory and learning were measured by the Novel Object Recognition (NOR) test found to improve in the rat group that received vitamin E.	Not Applicable	Vitamin E can improve learning and memory.
Iuliano et al.	2010	Case-control, experimental research	An enzymatic relationship between oxysterols (24S-hydroxycholesterol and 27 hydroxycholesterol, free radical related oxysterols of oxidative stress and Vitamin E were compared between 37 patients of Alzheimer’s disease, 24 MCI, 29 multi-domains (md-MCI).	People with mild cognitive impairment with oxidative stress found to be lower in Vitamin E.	Mini Mental State Examination, Mental Deterioration Battery (MDB)	Vitamin E might have some role in reducing oxidative stress delay and cognitive delay.
Whitehair et al.	2010	Experimental research design	The relationship of Apolipoprotein E ɛ4 (*APOE* ɛ4) allele was observed for 36 months of the period in 516 MCI patients age between 55 to 90 years who were on Vitamin E in 516 MCI participants aged 55–90 years who received placebo.	Vitamin E did not find to be associated with the progression of Apolipoprotein E ɛ4.	Mini Mental State Examination, Alzheimer’s Disease Assessment Scale-Cognitive subscale (ADAS-cog)	A direct connection between Vitamin E and the decline of cognitive function could not be found. However, the active status of *APOE* ɛ4 was found associated with a fast decline in cognitive function.
Mangialasche et al.	2012	Clinical study	This study examined the relationship between 8 natural compounds of Vitamin E with cognitive impairment. 166 MCI subjects were compared with cognitively normal people.	Low plasma tocopherols and tocotrienols levels of vitamin E were found with increased odds of MCI in people with ID.	The Folstein Mini-Mental State Examination (MMSE), Clinical dementia rating scale and Hachinski ischemic scale	Vitamin E may have a role against the progression of MCI to AD.
Mangialasche et al.	2013	Cohort research design	140 non-cognitively impaired people were observed for 8 years. The baseline serum vitamin E and cognitive impairment were observed.	The risk of cognitive impairment was found lower among those who had a moderate level of tocopherol/cholesterol ratio than those who had the lowest level of tocopherols.	Mini-Mental State Examination (MMSE)	Vitamin E might play an important role in cognitive impairment in humans. Vitamin E’s therapeutic role should be explored.
Shahar et al.	2013	Cross-sectional.	The relationship between MCI and Vitamin A and E was explored in a total of 333 participants age 60 years and above.	Vitamin E level was found lower in APOEe4 carriers that affect MCI.	The Folstein Mini-Mental State Examination (MMSE)	The role of vitamin E needs to be further explored in relation to MCI.
Dysken et al.	2014	Double-blind, placebo-controlled, parallel-group, randomized clinical trial	The effect of vitamin E on the progression of cognitive impairment was examined. 613 patients were recruited. They received either 2000 IU/d of alpha-tocopherol (*n* = 152), 20 mg/d of memantine (*n* = 155), the combination (*n* = 154), or placebo (*n* = 152).	Activities of Daily Living (ADCS-ADL) Inventory score declined in the group that was given vitamin E.	Activities of Daily Living (ADCS-ADL) Inventory score, Mini-Mental State Examination (MMSE)	Vitamin E can slow down the progression of cognitive impairment.
Zanotta, Puricelli & Bonoldi	2014	Prospective cohort	The effect of vitamin E in improving cognition in people diagnosed with MCI was assessed. 104 people about 70 years old were included in the research.	Vitamin E as a supplementary dietary found to be counteractive to cognitive impairment.	Alzheimer’s Disease Assessment Scale-Cognitive subscale (ADAS-cog)	Vitamin E may have a role in lowering the risk of MCI.
Naeini et al.	2014	Double-blind randomized, placebo-controlled trial	256 elderly, ages between 65 to 75 were received 300 mg vitamin E with 400 mg of vitamin C or placebo for 1 year	Vitamin E reduced the malondialdehyde level and raised total antioxidant capacity and glutathione.	Mini-Mental State Examination (MMSE)	Vitamin E supplementation did not appear to be enhancing cognitive performance
Li et al.	2015	Prospective cohort	This study examined the effect of vitamin E and C together and both vitamins independently on cognitive functions in the elderly population. 276 elderly people received Vitamin E and C together and E independently.	Radioimmunoassay (RIA) results, MMSE, and HDS assessments indicated improvement in cognitive functions with vitamin E and also when vitamin E was given in combination with Vitamin C.	Mini-mental state examination and Hasegawa Dementia Scale	Vitamin E can improve cognitive functions in the elderly population.
De Beaumont et al.	2016	Experimental research	The relationship of apolipoprotein E4 (APOE-ɛ4) gene and butyryl cholinesterase (BCHE) was assessed on the effect of cognitive impairment.	The study did not mention vitamin E; however, it was designed on the premise of that lower levels of vitamin E increases apolipoprotein E4 (APOE-ɛ4) and butyryl cholinesterase (BCHE) activity that increases declines memory.	histopathological confirmation of AD according to NINCDS-ADRDA criteria	Vitamin E may have a role in lowering the rate of memory impairment.
Basambombo et al.	2017	Cohort research design	The effect of Vitamin E and also Vitamin C was observed in a cohort of 5269 individuals aged 65 years and above in the Canadian Study of Health and Aging (1991–2002).	The baseline memory and learning ability were compared on the same standardized tests. Vitamin E and C together and independently were found to be associated with a lower risk of memory decline.	Modified Mini-Mental State (3MS) Examination.	Vitamin E plays a role in reducing the risk of memory decline in individuals.
Liu et al.	2018	Randomized controlled study	A randomized controlled study in 7781 individuals of European descent.	No association was observed between dietary supplementation of vitamin E with cognitive impairment.	Not available, since this study utilized biomarkers	The study suggests no association between vitamin E supplementation and MCI in the general population.
Edmonds et al.	2018	Experimental research design	The effect of donepezil and Vitamin E was compared for 756 MCI participants.	The donepezil treatment group had a lower rate of progression from MCI to AD than the Vitamin E group.	The Wechsler Memory Scale–Revised Logical Memory II subtest, Mini-mental state examination.	Vitamin E may not have an effect on lowering the rate of MCI towards AD.
Kim et al.	2018	Cross-sectional	The effect of serum vitamin A, C, and E was evaluated for the risk of cognitive impairment in 230 participants aged 60 to 79 years.	Association between vitamin A and C serum was not observed while a negative relationship between vitamin E, beta-gamma tocopherol was observed with a lower risk of cognitive impairment.	Korean version of the Mini-Mental State Examination	Serum beta-gamma tocopherol levels tended to be inversely associated with the risk of cognitive impairment.
Casati et al.	2019	Experimental research design	The relationship between Vitamin E forms and leukocyte telomere length (LTL) in AD was explored for the purpose of knowing its effect on MCI. Vitamin E forms (α-, β-, γ- and δ-tocopherol, α-, β-, γ- and δ-tocotrienol), the ratio of α-tocopherylquinone/α-tocopherol and 5-nitro-γ-tocopherol/γ-tocopherol (markers of oxidative/nitrosative damage) and LTL were measured in 53 AD subjects and 40 cognitively healthy controls (CTs).	People suffering from AD found to have lower concentrations of α-, β-, γ- and δ-tocopherol, α- and δ-tocotrienol, total tocopherols, total tocotrienols, and total vitamin E compared to CTs.	Not available, since this study utilized telomere length as an indicator	The study suggests that Vitamin E deficiency may be playing a role in AD pathology in progressing MCI to AD.
